# The role and mechanism of action of miR-483-3p in mediating the effects of IGF-1 on human renal tubular epithelial cells induced by high glucose

**DOI:** 10.1038/s41598-024-66433-y

**Published:** 2024-07-07

**Authors:** Maidina Abudoureyimu, Talaiti Tayier, Ling Zhang

**Affiliations:** 1 First Department of Comprehensive Internal Medicine of People’s Hospital of Xinjiang Uygur Autonomous Region, No.91 Tianchi Road, Urumqi, 830001 Xinjiang China; 2 Department of Urology, People’s Hospital of Xinjiang Uygur Autonomous Region, No.91 Tianchi Road, Urumqi, 830001 Xinjiang China

**Keywords:** miR-483-3p, IGF-1, High glucose, Renal tubular epithelial cells, Kidney injury, Cell biology, Diseases, Pathogenesis

## Abstract

This study aimed to elucidate the influence of miR-483-3p on human renal tubular epithelial cells (HK-2) under high glucose conditions and to understand its mechanism. Human proximal tubular epithelial cells (HK-2) were exposed to 50 mmol/L glucose for 48 h to establish a renal tubular epithelial cell injury model, denoted as the high glucose group (HG group). Cells were also cultured for 48 h in a medium containing 5.5 mmol/L glucose, serving as the low glucose group. Transfection was performed in various groups: HK-2 + low glucose (control group), high glucose (50 mM) (HG group), high glucose + miR-483-3p mimics (HG + mimics group), high glucose +miR-483-3p inhibitor (HG + inhibitor group), and corresponding negative controls. Real-time quantitative polymerase chain reaction (qPCR) assessed the mRNA expression of miR-483-3p, bax, bcl-2, and caspase-3. Western blot determined the corresponding protein levels. Proliferation was assessed using the CCK-8 assay, and cell apoptosis was analyzed using the fluorescence TUNEL method. Western blot and Masson’s staining were conducted to observe alterations in cell fibrosis post miR-483-3p transfection. Furthermore, a dual-luciferase assay investigated the targeting relationship between miR-483-3p and IGF-1. The CCK8 assay demonstrated that the HG + mimics group inhibited HK-2 cell proliferation, while the fluorescent TUNEL method revealed induced cell apoptosis in this group. Conversely, the HG + inhibitor group promoted cell proliferation and suppressed cell apoptosis. The HG + mimics group upregulated mRNA and protein expression of pro-apoptotic markers (bax and caspase-3), while downregulating anti-apoptotic marker (bcl-2) expression. In contrast, the HG + inhibitor group showed opposite effects. Collagen I and FN protein levels were significantly elevated in the HG + mimics group compared to controls (P < 0.05). Conversely, in the HG + inhibitor group, the protein expression of Collagen I and FN was notably reduced compared to the HG group (P < 0.05). The dual luciferase reporter assay confirmed that miR-483-3p could inhibit the luciferase activity of IGF-1’s 3′-UTR region (P < 0.05). miR-483-3p exerts targeted regulation on IGF-1, promoting apoptosis and fibrosis in renal tubular epithelial cells induced by high glucose conditions.

## Introduction

Diabetic kidney disease (DKD) is a progressively debilitating renal condition that constitutes a substantial health concern, affecting an estimated 40% of individuals with diabetes mellitus. It ranks as one of the leading causes of End-Stage Renal Disease (ESRD), imposing a considerable burden on healthcare systems worldwide^1^.

DKD is characterized by a multifaceted pathogenesis involving a complex interplay of genetic, environmental, and metabolic factors. Pervious evidence has underscored the role of renal tubulointerstitial fibrosis in driving the progression of DKD^2^. However, the precise molecular underpinnings of this phenomenon remain unclear.

As a class of small non-coding RNA molecules, microRNA (miRNA), have emerged as key regulators of gene expression in various cellular processes. In recent years, miRNAs have garnered increasing attention due to their ability to fine-tune intricate signaling networks, modulating cell behavior and ultimately influencing disease progression. Recent studies have shown that miR-483-3p has a clear anti-fibrotic effect^3,4^, and the increased level of miR-483-3p can impair the activity of endothelial cells induced by high glucose^5^. Besides, another study reported that miR-483-3p has been found to be abnormally expressed in DKD in vitro and in vivo models^6^. Nevertheless, the role of miR-483-3p in diabetic kidney disease (DKD) and its underlying molecular mechanisms remain unclear.

Insulin-like growth factor-1 (IGF-1) is a growth factor crucial for maintaining the structure and function of glomeruli and renal tubular cells, playing a pivotal role in the pathogenesis of diabetic nephropathy (DN). Previous studies have demonstrated that the overexpression of IGF-1 can induce renal tissue hyperplasia, renal cell proliferation, renal hypertrophy, renal mesangial dilation, and an increase in the expression of inflammatory factors and extracellular matrix (ECM) proteins. Simultaneously, studies have reported that IGF-1, along with its receptors and downstream pathways (PI3K-Akt pathway and ERK pathway), exerts a functional role in modulating cell proliferation, apoptosis, and fibrosis in HK-2 cells. At the same time, it has been reported that IGF-1 and its receptors and downstream pathways (PI3K-Akt pathway and ERK pathway) can play a functional role in regulating cell proliferation, apoptosis and fibrosis in HK-2 cells^7,8^.

This study established a diabetic kidney disease model using high glucose-induced human renal tubular epithelial cells, and explore the molecular mechanisms of renal tubular epithelial cell injury induced by high glucose through miR-483-3p. This work aims to provide a theoretical foundation for a deeper understanding of the intricate molecular mechanisms involved in renal tubular epithelial cell injury.

## Materials and methods

### Cell lines

Human renal tubular epithelial cells HK-2 were sourced from the Chinese collection center for exemplary cultures at Wuhan University. And these cells were cultivated in a culture medium comprising Dulbecco's Modified Eagle Medium/Nutrient Mixture F-12 (DMEM/F12) supplemented with 10% fetal bovine serum. The cellular cultivation was conducted within a controlled environment maintained at a temperature of 37 °C, under an atmospheric composition of 5% carbon dioxide, and within conditions of saturated humidity, within a cell culture incubator.

### Cell resuscitation and passage

Extract the HK-2 cells from the liquid nitrogen repository, swiftly placing them into a 37 °C water bath. Gently agitate the cryogenic storage vial to facilitate the dissolution of the cryopreservation solution. Subsequent to dissolution, transfer the cells into a centrifuge tube containing 5 ml of culture medium (comprising DMEM + 10% FBS + 1% Penicillin–Streptomycin solution).

Suspend the cells in a complete growth medium containing 10% fetal bovine serum, subsequently inoculating them into a culture dish. Gently tap and agitate the dish to ensure homogenous distribution. Maintain cultivation under conditions of 37 °C, 5% CO2 saturation, and humidity. Upon achieving a cellular density of 80%, initiate subculturing.

Subsequently, add 1–2 ml of 0.25% trypsin to enzymatically digest the cells. Observe under a microscope as the cells undergo digestion, which usually takes 30–60 s, leading to their detachment and rounding–indicative of the completion of digestion. Supplement with a complete growth medium and perform mechanical agitation to produce a single-cell suspension.

### Cell transfection treatment

The pYr-Mir Target vector was procured from YRBio. Select HK-2 cells in the logarithmic growth phase, characterized by robust growth status. Plate them at a density of 5 × 10^3^ cells per well in a 96-well plate, concurrently establishing a control group with no treatment. Prior to transfection, pre-condition the cells by culturing them in serum-free DMEM medium for 2 h. Take 50 ml of complete medium, sequentially add 50, 150, 250, and 350 mg of D-glucose hydrate, to prepare high-glucose media with concentrations of 11.1, 22.1, 33.3, and 44.4 mmol/L, respectively. Adjust the osmolarity with D-mannitol, mix thoroughly, seal, and store at 4 °C for future use. Employ the following categorizations for transfection: Control (low glucose group), high glucose (50 mM) (HG group), high glucose + mimics of non-coding sequence (HG + mimics NC group), high glucose + miR-483-3p mimics (HG + mimics group), high glucose + non-coding sequence inhibitor (HG + inhibitor NC group), high glucose + miR-483-3p inhibitor (HG + inhibitor group).

Transfection: 1 μg of siRNA (concentration 20 μM) was diluted in 10 μl of Opti-MEM (GIBCO, 51985-034) without serum, mixed gently with a pipette, and allowed to incubate at room temperature for 5 min. Prior to use, LipofectamineTM 2000 (Invitrogen, 52887) was gently mixed. Then, 0.5 μl of Lipofectamine TM 2000 was diluted in 10 μl of Opti-MEM and incubated at room temperature for 5 min. After a 5 min incubation, the mixture of LipofectamineTM 2000 and siRNA (total volume 20 μl) was gently combined and incubated at room temperature for an additional 20 min.Add 20 μl of the mixed transfection solution into each well, gently tilting the cell culture plate to ensure even distribution. Incubate the cells at 37 °C in a CO2 incubator. After 6 h, carefully aspirate the transfection mixture and replace it with regular culture medium. Continue culturing at 37 °C, 5% CO_2_ for 48 h. Following a 2 h incubation with 0.1 μM Ferrostatin-1, proceed with subsequent assessments.

### CCK8 detection

Following a 48 h cell cultivation period as described above, introduce 10 μL of CCK8 solution into each well. Incubate the plate at 37 °C for 2 h. Subsequently, remove the culture medium and employ an enzyme-linked immunosorbent assay (ELISA) reader to determine the optical density (OD) at 450 nm for each well.

### Apoptosis detection

Utilize a 0.25% trypsin solution devoid of EDTA to enzymatically digest the cells. Employ the Annexin V-APC/7-AAD apoptosis detection kit for assessment. Mix 5 μL of 7-AAD dye solution with 50μL of Binding Buffer. Allow the mixture to react in a light-protected environment at room temperature for 5 to 15 min. Post-reaction, add 450 μL of Binding Buffer to the mixture and mix well. Incorporate 5 μL of Annexin V-APC, ensuring proper mixing, and subsequently allow the reaction to occur in a light-protected environment at room temperature for 5 to 15 min. Analyze the samples using a flow cytometer.

### Tunel

Immerse the cellular slides into a 4% solution of paraformaldehyde (pH 7.4) at room temperature for a duration of 15 min to perform fixation. Subsequently, perform a series of three PBS washes, each lasting 5 min. Employ a 0.2% Triton X-100 (prepared in PBS) solution at room temperature for a permeabilization period of 20 min. Submerge the adequately permeabilized samples in PBS for a sequence of three 5 min rinses. Post-treatment, store the samples in a humidified container to ensure their preservation.

For nuclear counterstaining, complete the incubation and perform three PBS washes for each sample, lasting 5 min each. After blotting excess moisture with absorbent paper, add the appropriately diluted DAPI solution and incubate at room temperature, protected from light, for 5 min. Subsequent to this incubation, perform another sequence of three PBS washes for each sample, lasting 5 min each. Carefully blot any liquid from the slides with absorbent paper. Seal the slides with a mounting medium containing fluorescence-quenching agents. Finally, employ a fluorescence microscope to observe and capture images.

### Masson staining

Within the culture dish, gently immerse the cell-seeded coverslips in PBS for three cycles, each spanning a minute. Proceed with fixation by submerging the coverslips in a 4% paraformaldehyde solution for 15 min. Rinse the coverslips with PBS thrice, each rinse extended to a duration of three minutes.

Engage in Weigert’s iron hematoxylin staining solution for a nucleus staining period of 5 min. Subsequently, execute a thorough water rinse for several minutes. Employ a 1% hydrochloric acid alcohol differentiation for a brief span of 3 s, followed by a comprehensive water rinse spanning 5 min.

Perform a 1-min rinsing step with 1% acetic acid after which, engage in rapid water rinsing. Perform dehydration using 95% ethanol, followed by absolute ethanol, and ultimately xylene. Following dehydration, employ neutral mounting medium for slide sealing. Conclude the process with microscopic examination.

### Quantitative real-time polymerase chain reaction (qRT-PCR)

Utilizing the DNAMAN software, the primers for miR-483-3p, IGF1, bax, bcl-2, and caspase-3 were meticulously designed. A mixture comprising 4 µL of 4 × gDNA Wiper Mix, 2 µg of Total RNA, and RNase-free ddH2O was brought to a total volume of 16 µL and gently mixed. After a brief centrifugation, the mixture was subjected to a thermal incubation at 42 °C for 2 min using a PCR instrument. Information of primer sets for qPCR showed in Table 1.

**Table 1:** The information of primer sets for qPCR.

Gene	Primer	Sequence (5′-3′)
Homo GAPDH	Forward	TCAAGAAGGTGGTGAAGCAGG
	Reverse	TCAAAGGTGGAGGAGTGGGT
U6	Forward	CGCTTCGGCAGCACATATAC
	Reverse	AAATATGGAACGCTTCACGA
miR-483-3p	loop primer	GTCGTATCCAGTGCAGGGTCCGAGGTATTCGCACTGGATACGACAAGACGGG
	F primer	TGCGCTCACTCCTCTCCTCCC

Gene	Primer	Sequence (5′-3′)
Homo GAPDH	Forward	TCAAGAAGGTGGTGAAGCAGG
	Reverse	TCAAAGGTGGAGGAGTGGGT
Homo IGF1	Forward	TCATGCCTTGGTCTCCTTGT
	Reverse	TGCTTTGATGGTCAGGTTGC
Homo Bax	Forward	AAGAAGCTGAGCGAGTGTCT
	Reverse	GTTCTGATCAGTTCCGGCAC
Homo bcl-2	Forward	TGTGGAGAGCGTCAACCG
	Reverse	AGACAGCCAGGAGAAATCAAA
Homo caspase-3	Forward	AGAGGGGATCGTTGTAGAAGTC
	Reverse	ACAGTCCAGTTCTGTACCACG

The qPCR reaction commenced with an initial denaturation step at 95 °C for 10 min, followed by 40 cycles consisting of denaturation at 95 °C for 15 s, annealing at 60 °C for 60 s, and extension at 95 °C for 15 s. Subsequently, a melt curve analysis was conducted at 60 °C for 1 min, followed by denaturation at 95 °C for 15 s. These steps culminated in the acquisition of qPCR reaction results, which were quantified using the 2^-ΔΔCt method.

### Western blot

Employing the Western blot technique, alterations in the protein expression levels of bax, bcl-2, caspase-3, and Cleaved-caspase-3 were ascertained. The procedure entailed the lysis and centrifugation of cellular samples from different experimental groups, followed by protein extraction and quantification using the BCA method.

The transferred membranes were immersed in a 5% TBST, 5% skimmed milk powder (blocking solution), blocking solution and incubated on a laboratory shaker for 2 h at room temperature. Subsequently, the membranes were subjected to visualization, and the developed films were analyzed using BandScan to quantify the intensity ratio (expressed as a percentage) between the target protein bands and reference protein bands.

### Dual luciferase reporter gene detection

Upon complete aspiration of the cellular culture medium, introduce 300 µL of cell lysis buffer for reporter genes. After thorough lysis, centrifuge at 15,000 rpm for 3 min, and collect the supernatant for subsequent analysis. Thaw the firefly luciferase assay reagent and Renilla luciferase assay detection buffer, and allow them to equilibrate to room temperature.

For each sample requiring 100 µL, take an appropriate amount of Renilla luciferase assay detection buffer, and prepare the Renilla luciferase assay working solution by adding Renilla luciferase assay substrate (100X) at a 1:100 ratio. Take 100 µL of the sample, add 100 µL of firefly luciferase assay reagent, and after thorough mixing, measure the relative luminescence units (RLU). Subsequently, add 100 µL of the Renilla luciferase assay working solution, mix well, and measure the RLU. Finally, with firefly luciferase as the internal control, calculate the ratio by dividing the RLU value obtained from the Renilla luciferase assay by the RLU value obtained from the firefly luciferase assay.

### Statistical analysis

Utilizing SPSS 25.0 software, data manipulation and analysis were conducted. All data were presented as means ± standard deviation (x̄ ± s). For quantitative variables, one-way analysis of variance (ANOVA) was employed, followed by the Least Significant Difference (LSD) method for pairwise comparisons. The significance level was set at α = 0.05, and statistical significance was determined when P < 0.05.

## Results

### miR-483-3p inhibits cell proliferation and induces apoptosis

**Figure 1:**
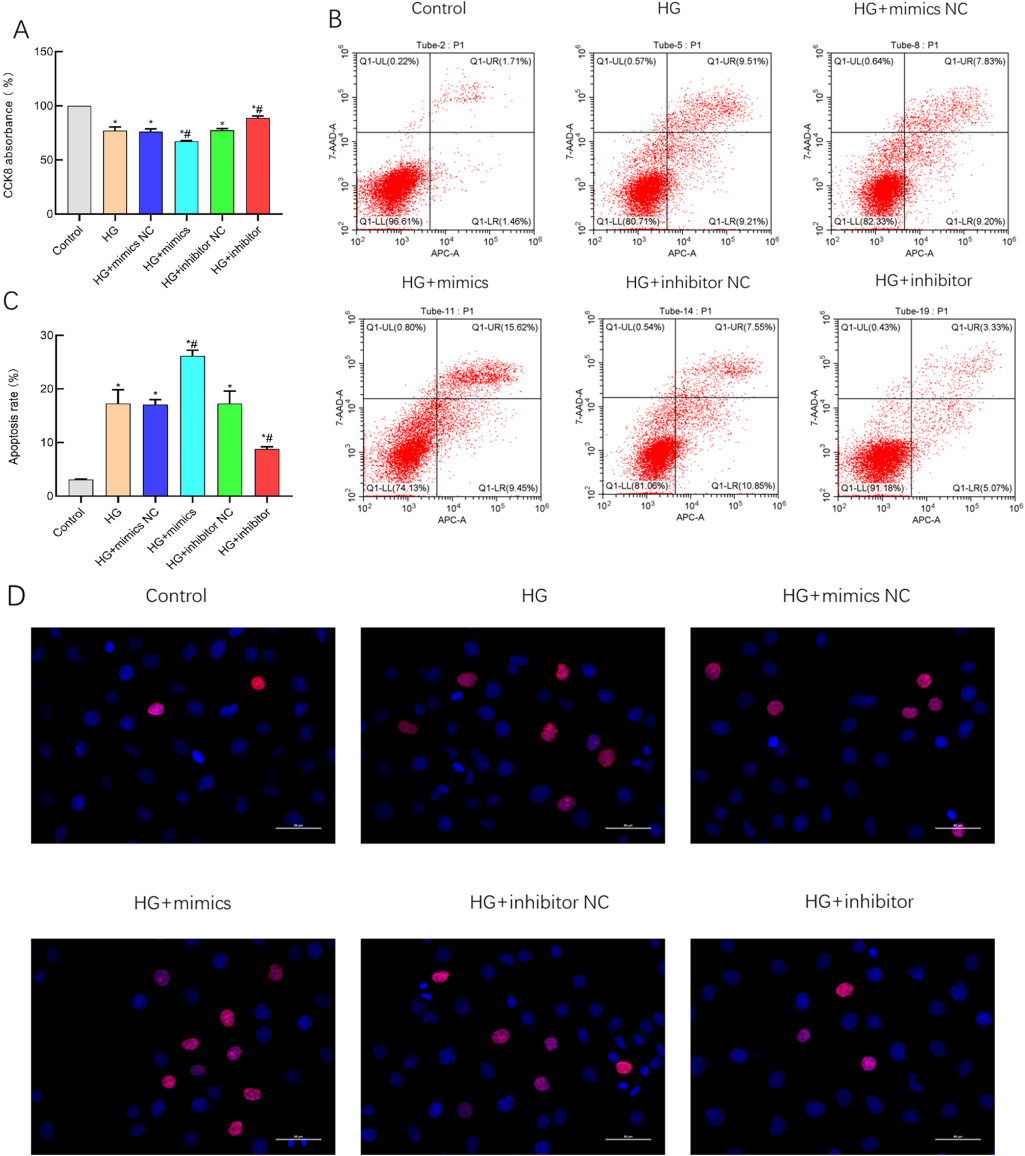
Suppression of cell proliferation and induction of apoptosis by miR-483-3p. (**A**) Analysis results from CCK-8 assay; (**B**) Results of flow cytometry apoptosis detection; (**C**) Flow cytometry apoptosis analysis chart; (**D**) Original Tunel image. *represents significant difference from the Control group, P < 0.05; #represents significant difference from the HG group, P < 0.05.

**Figure 2:**
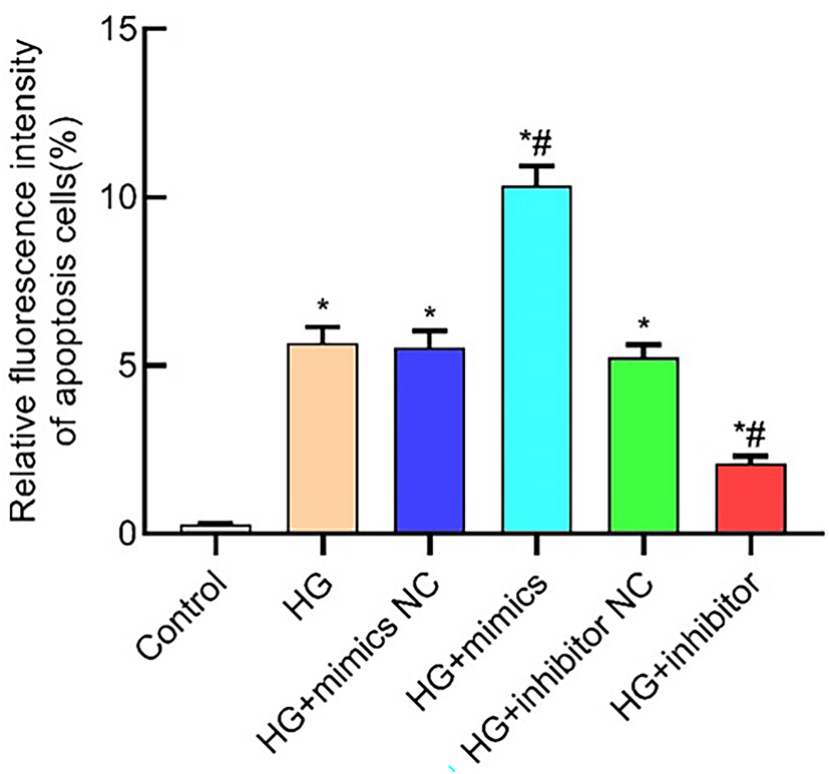
Analysis result of tunel image.

The experimental findings from the CCK-8 assay reveal that the HG + mimics group inhibits the proliferative activity of HK-2 cells (Fig. 1A). Flow cytometry analysis of cellular apoptosis indicates a significant increase in the induction of apoptosis in the HG + mimics group compared to the control group (Fig. 1B,C), The apoptosis rate (%) = Q1-UR (%) + Q1-LR (%). Tunel assay results also demonstrate an elevated induction of cellular apoptosis in the HG + mimics group, which is counteracted upon the addition of a miR-483-3p-specific inhibitor (Fig. 1D). These data collectively underscore that miR-483-3p can reduce the cell viability of HK-2 cells under the condition of high glucose, resulting in decreased cell proliferation, and thus promote the progression of apoptosis (Fig. 2).

### miR-483-3p regulates apoptosis-related factors

**Figure 3:**
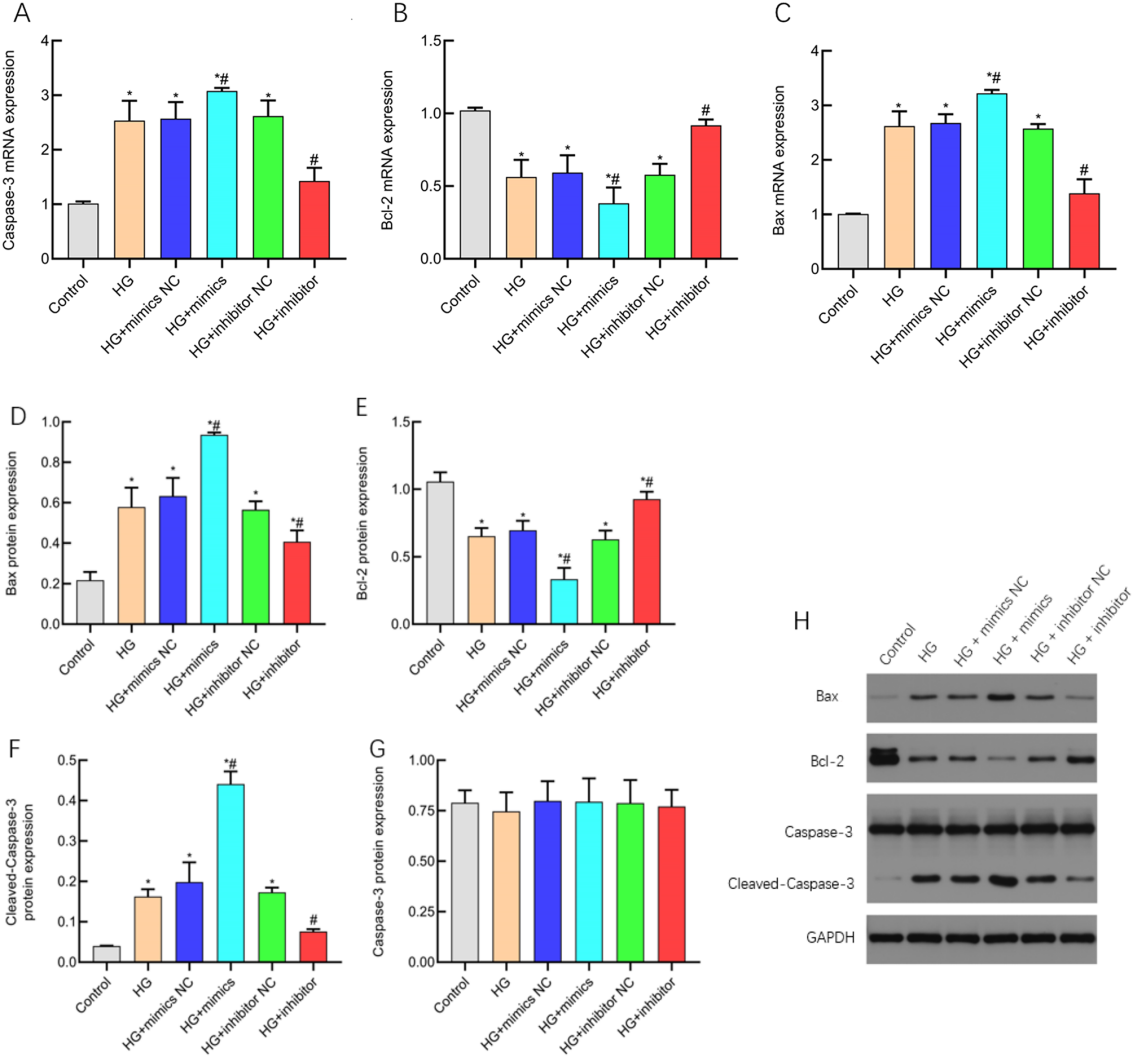
miR-483-3p induces the expression of pro-apoptotic factors, suppresses the expression of anti-apoptotic factors, and promotes cellular apoptosis. The horizontal axis represents cellular groups, and the vertical axis represents molecular expression levels. (**A**) Analysis of caspase-3 mRNA; (**B**) Analysis of bcl-2 mRNA; (**C**) Analysis of bax mRNA; (**D**) Analysis of bax protein; (**E**) Analysis of bcl-2 protein; (**F**) Analysis of cleaved-caspase-3 protein; (**G**) Analysis of caspase-3 protein; (**H**) Original western bolt image. *represents significant difference from the control group, P < 0.05; #represents significant difference from the HG group, P < 0.05.

Utilizing qPCR and Western blot analysis, the post-transfection state of miR-483-3p and the expression levels of bax, bcl-2, and caspase-3 were assessed. The findings of this investigation indicate that the HG + mimics group upregulated the expression of caspase-3 and bax mRNA (Fig. 3A,C), concomitantly promoting the protein expression (Fig. 3D,F,G). The HG + mimics group also attenuates the mRNA and protein expression of bcl-2 (Fig. 3B,E). In contrast, the HG + inhibitor group exhibited suppression of bax and caspase-3 mRNA expression, leading to reduced protein levels of bax and cleaved-caspase-3, along with the augmentation of bcl-2 expression. These observations (Supplementary Materials) collectively suggest that miR-483-3p orchestrates apoptosis in HK-2 cells by enhancing the expression of pro-apoptotic factors and dampening the expression of anti-apoptotic factors (Fig. 3H).

### miR-483-3p induces fibrosis in HK-2 cells

**Figure 4:**
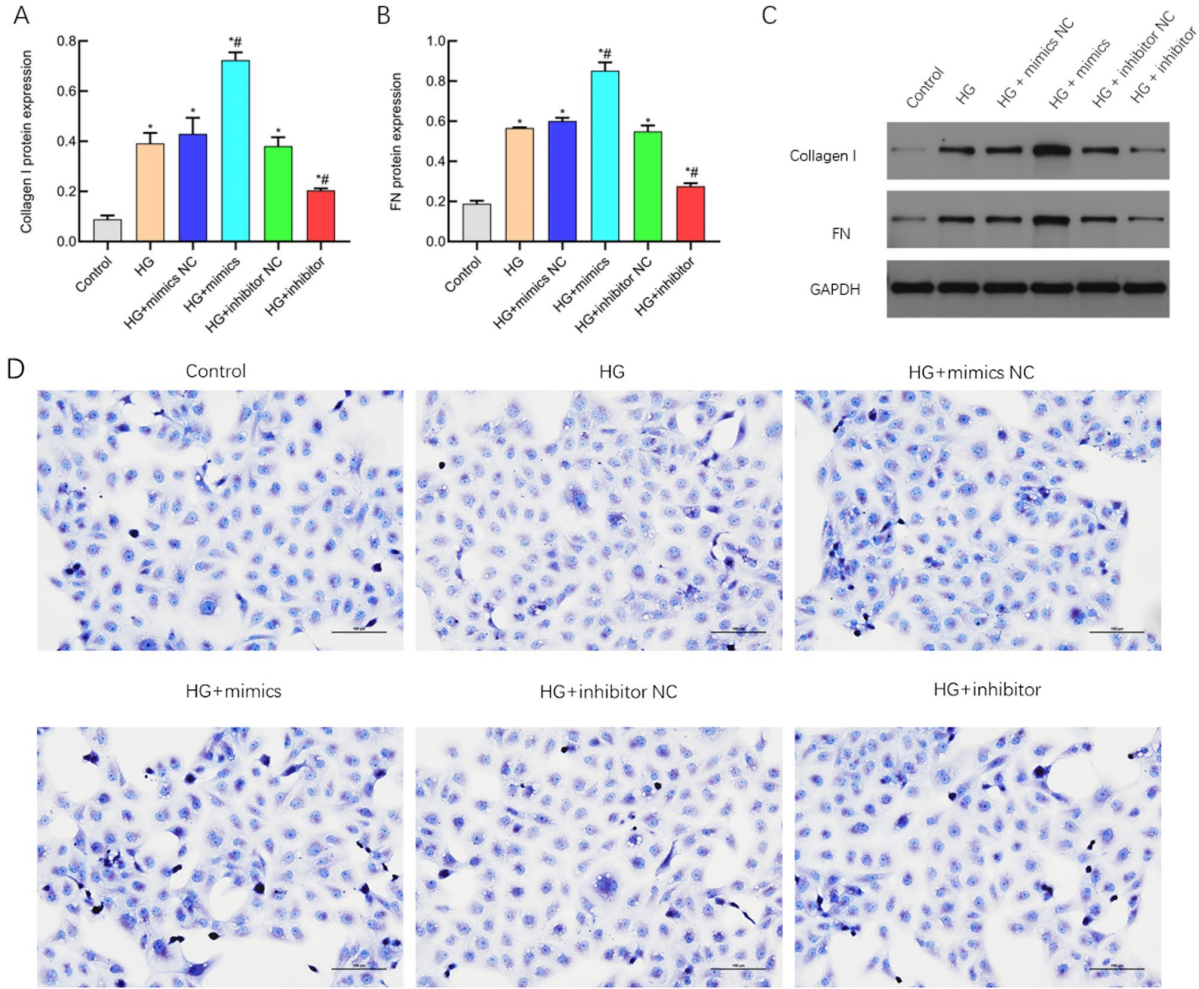
miR-483-3p induced fibrosis in HK-2 Cells. X-axis represents cell groups; Y-axis represents molecular expression levels. (**A**) Collagen I protein analysis chart; (**B**) E-cadherin protein analysis chart; (**C**) Original western blot image; (**D**) Original Masson’s staining image. *represents significant difference from the control group, P < 0.05; #represents significant difference from the HG group, P < 0.05.

Subsequently, we employed Western blotting and Masson’s trichrome staining to elucidate the alterations in cellular fibrogenesis following miR-483-3p transfection in HK-2 cells. The research findings reveal a substantial elevation in the protein expression levels of Collagen I and FN in the HG + mimics group compared to both the Control and HG groups (P < 0.05). Conversely, the HG + inhibitor group displayed a notable reduction in the protein expression levels of Collagen I and FN in contrast to the HG group (P < 0.05) (Fig. 4A,C). FN is a chemokine for fibroblast growth and promotes fibrosis formation (Fig. 4B). Masson’s trichrome staining exhibited enhanced positivity for collagen fibers in the HG + mimics group (intensified bluecoloration), whereas the HG + inhibitor group showed diminished collagen fiber positivity (reduced blue staining) (Fig. 4D). These findings collectively signify that miR-483-3p is capable of augmenting the expression of Collagen I and FN, thereby fostering fibrogenic processes in HK-2 cells.

### Biological functions of miR-483-3p are mediated by IGF1

**Figure 5:**
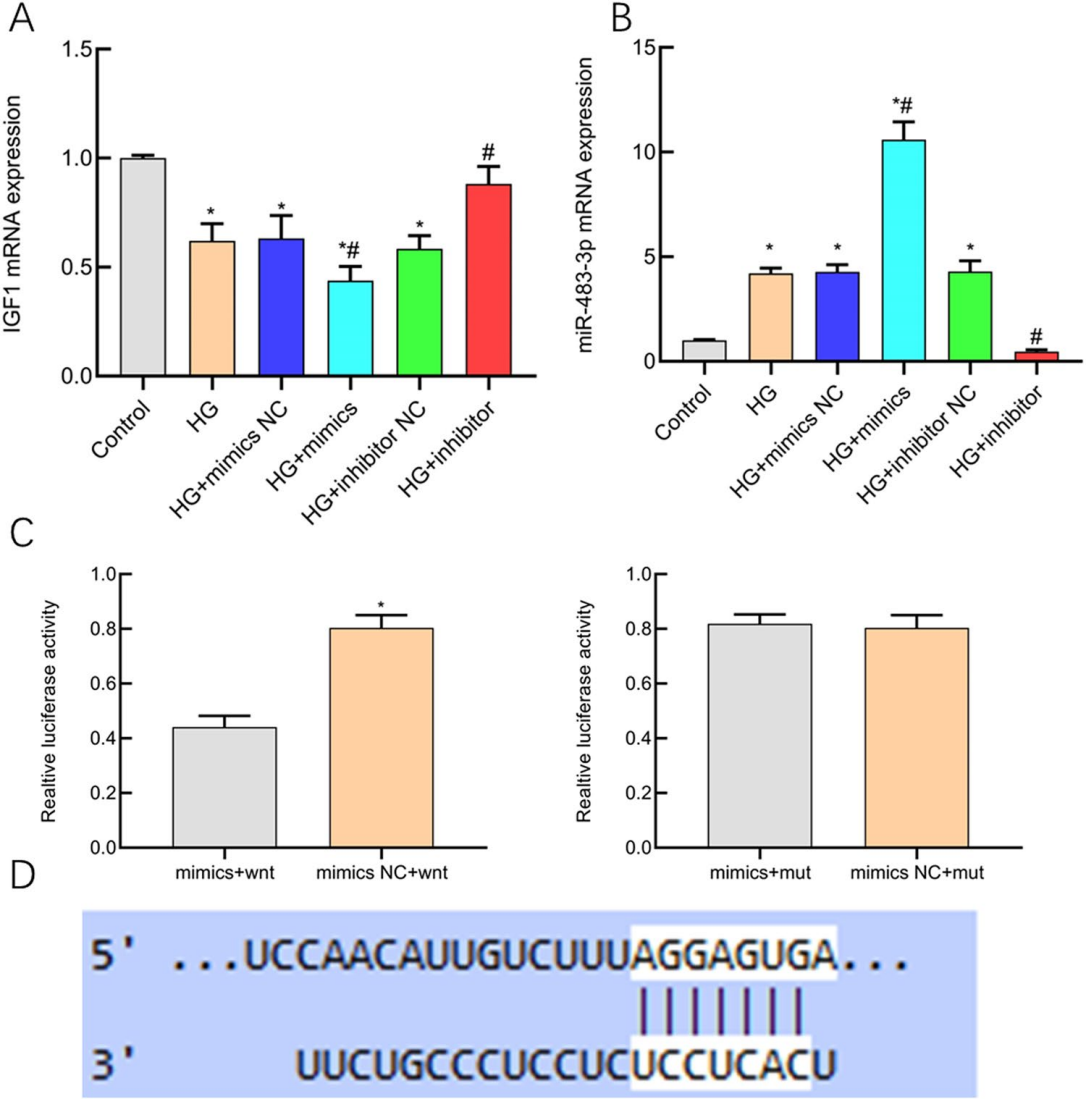
The study employed dual-luciferase reporter gene assays and real-time quantitative PCR to investigate the regulatory correlation between miR-483-3p and IGF1. The findings revealed that the miR-483-3p level in the HG + mimics group was significantly higher than in the control group and the HG group (P < 0.05), while IGF1 was significantly lower than in the control group and the HG group (P < 0.05). In the HG + inhibitor group, the miR-483-3p level was significantly lower than in the HG group (P < 0.05), and IGF1 was significantly higher than in the HG group (P < 0.05) (**A**,**B**). The dual-luciferase reporter gene results confirmed a negative regulatory relationship between miR-483-3p and IGF1 (P < 0.05) (**C**,**D**). These data suggest that miR-483-3p and IGF1 exhibit a targeted negative regulatory relationship.

Employing a dual luciferase reporter assay and qPCR, we have undertaken an investigation into the regulatory interrelation between miR-483-3p and IGF1. The outcomes of our inquiry have illuminated that within the HG + mimics cohort, miR-483-3p has exhibited a substantial elevation in contrast to both the control and HG groups (P < 0.05), whereas IGF1 has evinced a notable decline relative to the control and HG groups (P < 0.05). Conversely, within the HG + inhibitor assemblage, miR-483-3p has experienced a noteworthy reduction vis-à-vis the HG group (P < 0.05), accompanied by a pronounced escalation in IGF1 expression compared to the HG group (P < 0.05, Fig. 5A,B). The results stemming from the dual luciferase reporter gene assay have duly validated a discernible negative regulatory correlation between miR-483-3p and IGF1 (P < 0.05, Fig. 5C,D). In summary, these findings implied that the inhibition of HK 2 cell proliferation and the induction of apoptosis by miR-483-3p might be mediated through IGF1.

### miR-483-3p plays a biological role in regulating TGF-β/smad signaling pathway

**Figure 6:**
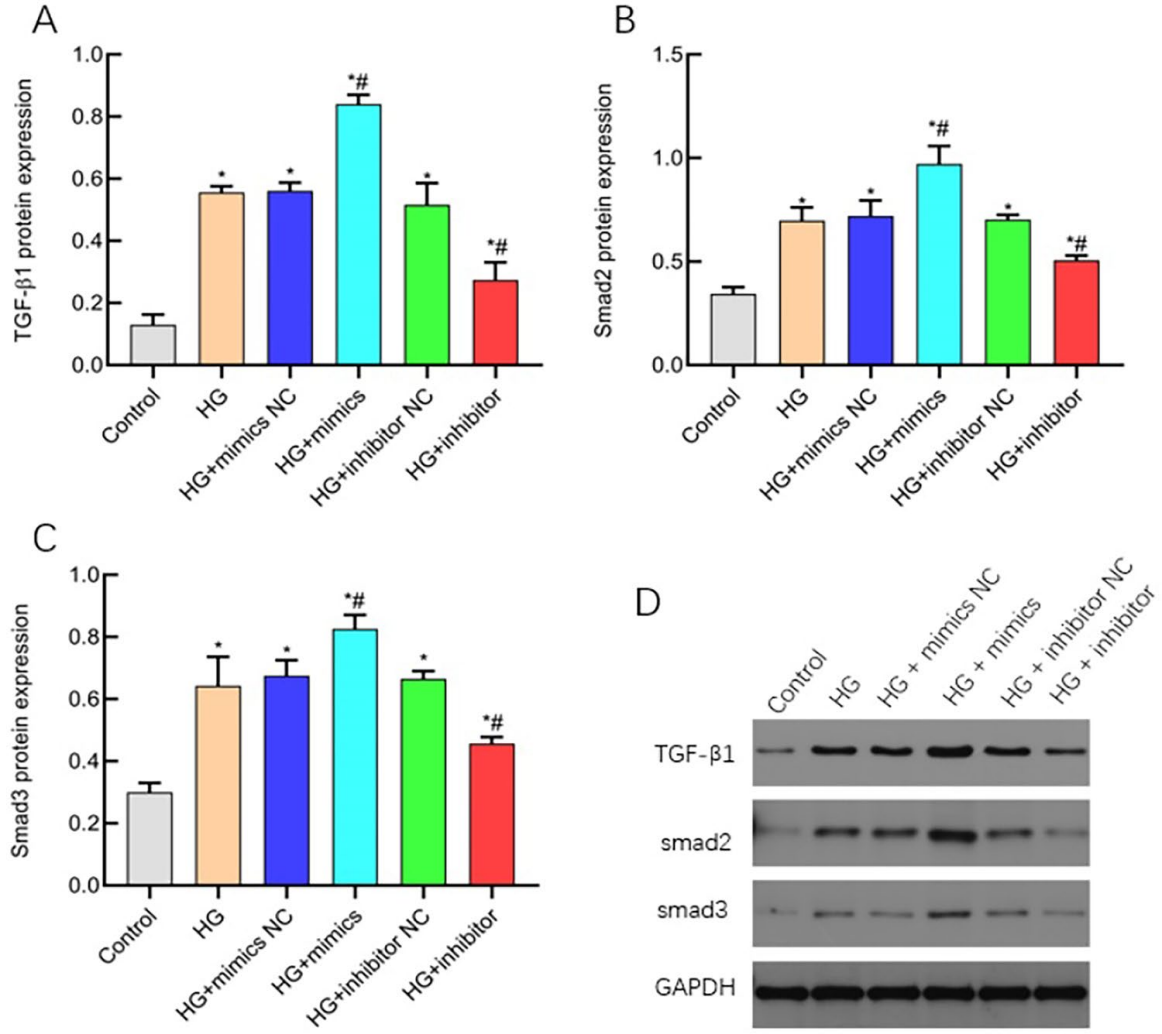
miR-483-3p plays a biological role in regulating TGF-β/smad signaling pathway. The horizontal coordinate is cell grouping, and the vertical coordinate is molecular expression. (**A**) TGF-β1 protein analysis; (**B**) Protein analysis of smad2; (**C**) smad3 protein analysis diagram; (**D**) Western blot of the original glue running. *indicates the difference between the Control group and the control group, P < 0.05; #indicates the difference between HG group and Hg group, P < 0.05.

Western blot was used to detect the protein expression of TGF-β1, smad2 and smad3 in TGF-β/smad signaling pathway. The results showed that the protein expressions of TGF-β1, smad2 and smad3 in HG + mimics group were significantly higher than those in control group and HG group (P < 0.05); The protein expressions of TGF-β1, smad2 and smad3 in HG + inhibitor group were significantly decreased compared with those in HG + mimics group (P < 0.05, Fig. 6A–D). These data indicate that miR-483-3p can activate TGF-β/smad signaling pathway activity and affect the occurrence and development of diseases.

## Discussion

The etiology of diabetic nephropathy remains enigmatic, compounded by a dearth of efficacious therapeutic interventions. Renal tubular lesions and interstitial fibrosis are pivotal elements in the pathogenesis of diabetic nephropathy, characterized by the deposition of extracellular matrix (ECM), predominantly composed of collagen and fibronectin. MiR-483-3p has been reported to exert a definitive antifibrotic role in liver fibrosis, exhibiting inhibitory effects on several fibrotic signaling pathways^6,9^. Nevertheless, the potential impact of miR-483-3p on renal fibrosis remains to be elucidated.

It has been reported in studies that miR-483-3p expression is upregulated in endothelial cells of diabetic patients^5^. In this study, we have confirmed an elevation in the expression of miR-483-3p in HK-2 cells subjected to high glucose treatment as compared to normal HK-2 cells. Furthermore, we investigated the impact of miR-483-3p upregulation on renal tubular epithelial cells and the underlying mechanisms. We did not observe the upregulation of miR-483-3p expression in human proximal tubular cells other than HK-2 cells, and we did not perform renal biopsies in patients with DKD (diabetic kidney disease). These experiments in future research to provide a more significant. The renal tubular epithelial cell (HK-2 cell) is among the most active cells in the tubulointerstitium and plays a crucial role in the physiological function and organizational structure of the kidney. In the presence of factors such as inflammation, hypoxia, and poisoning, which induce injury to the kidney, a series of changes occurs in the renal tubules. These changes include the activation and proliferation of renal tubule epithelial cells, leading to the expansion of the renal tubules. Eventually, these alterations can result in outcomes such as atrophy, apoptosis, and fibrosis of the renal tubules. Moreover, HK-2 cells have been widely utilized as a cell model for studying fibrosis in diabetic nephropathy in numerous research studies^10,11^.

Based on current knowledge, an increase in cell apoptosis usually occurs prior to renal fibrosis^12^, although the mechanisms triggering apoptosis and initiating renal fibrosis remain relatively underexplored. These mechanisms might involve inflammatory mediators, activation of the renin-angiotensin-aldosterone system, and epithelial-mesenchymal transition (EMT) of renal tubular epithelial cells^13–15^. Our research findings substantiate this hypothesis, indicating that the heightened levels of miR-483-3p promote the expression of apoptosis-inducing factors, while concurrently reducing the expression of anti-apoptotic factors, thus inducing apoptosis in HK-2 cells. Further analysis reveals that inhibiting miR-483-3p expression significantly suppresses high glucose-induced apoptosis in renal tubular epithelial cells, leading to reduced Caspase-3 expression levels. This suggests that the suppression of miR-483-3p expression may mitigate cell apoptosis by downregulating Caspase-3 expression, thereby alleviating injury to renal tubular epithelial cells induced by high glucose. Concurrently, overexpression of miR-483-3p results in upregulated expression of fibrosis markers, Collagen I and fibronectin (FN), promoting fibrogenesis in HK-2 cells. This indicates that high glucose-induced apoptosis in renal tubular epithelial cells may concurrently trigger interstitial fibrosis in the kidney. Studies have shown that apoptosis can lead to renal extracellular matrix deposition, tubular epithelial cell hypertrophy, and glomerular membrane dilation, leading to tubular interstitial fibrosis and glomerular sclerosis^16–19^.

Further analysis reveals that miR-483-3p can selectively target and bind to insulin-like growth factor I (IGF-I), thereby exerting a negative modulation on the expression of IGF-I. Findings from the realm of cancer biology suggest that apart from its role in transcriptional regulation alongside its host gene IGF-2, miR-483-3p may also engage in independent regulation through the WNT/β-catenin signaling pathway, distinct from IGF-2 transcriptional control^20,21^. Our investigations, however, were unable to validate this observation, possibly due to the context-specific differences manifested by the co-presence of basal target gene expression and shared transcriptional co-regulators.

This study has unveiled that overexpression of miR-483-3p inhibits the expression of IGF-1 protein, thereby promoting apoptosis in renal tubular epithelial cells and initiating renal fibrosis. Research indicates that IGF-1, as a vital growth factor required for normal renal development, can stimulate DNA synthesis within proximal tubular epithelial cells and renal glomerular mesangial cells, prompting cell proliferation and microvascular expansion to enhance glomerular filtration rate and renal plasma flow^22^. Animal studies have revealed that IGF-1 administration holds the potential to reduce tubular cell apoptosis, attenuate tubular atrophy, and counteract interstitial collagen accumulation, effectively mitigating interstitial fibrosis^23^. Consequently, IGF-1 emerges as a pivotal renal protective factor.

Research have shown that TGF-β1 and its downstream signaling pathways play a significant role in the pathogenesis of diabetic nephropathy^24^. TGF-β1 not only promotes the proliferation of mesangial cells, deposition of mesangial matrix, and glomerulosclerosis, but also leads to tubulointerstitial fibrosis in the kidneys^25^. As part of the TGF-β1 pathway, SMAD proteins, particularly SMAD2, SMAD3, SMAD4, and SMAD7, are crucial in diabetic nephropathy. In this study, we found that miR-483-3p can activate the TGF-β/smad signaling pathway, influencing the onset and progression of the disease. It has been shown that in certain tumor development or physiological processes, TGF-β1 and the IGF1 signaling pathways exhibit reciprocal regulation. For instance, Ochiai et al.^26^ found that persistently high expression of h-TGF-β1 can suppress the expression of IGF-1, thereby inhibiting osteoblast differentiation. Walsh LA et al. discovered that IGF-1 signaling can induce the activation of latent TGF-β1, leading to epithelial-mesenchymal transition in breast cancer cell lines^27^. Song et al.^28^ demonstrated that IGF-1 can inhibit TGF-β transcription by specifically suppressing the expression of Smad3. Furthermore, there is evidence that these two signaling pathways share a common downstream protein, AKT^26^.

In conclusion, the elevated levels of miR-483-3p impair the survival of renal tubular cells induced by high glucose, hinder the proliferation of renal tubular epithelial cells, and promote apoptosis. This consequently suppresses the regenerative capacity post-injury and accelerates the progression of fibrosis processes.

### Supplementary Information

Supplementary Information 1.

Supplementary Information 2.

Supplementary Information 3.

## Data Availability

The raw data can be made available upon reasonable request to the corresponding author.
