# Supplementary figures and images for: The role and mechanism of action of miR-483-3p in mediating the effects of IGF-1 on human renal tubular epithelial cells induced by high glucose
Source: Sci Rep. 2024 Jul 7;14:15635. doi: 10.1038/s41598-024-66433-y (PMC11228025; doi:10.1038/s41598-024-66433-y)

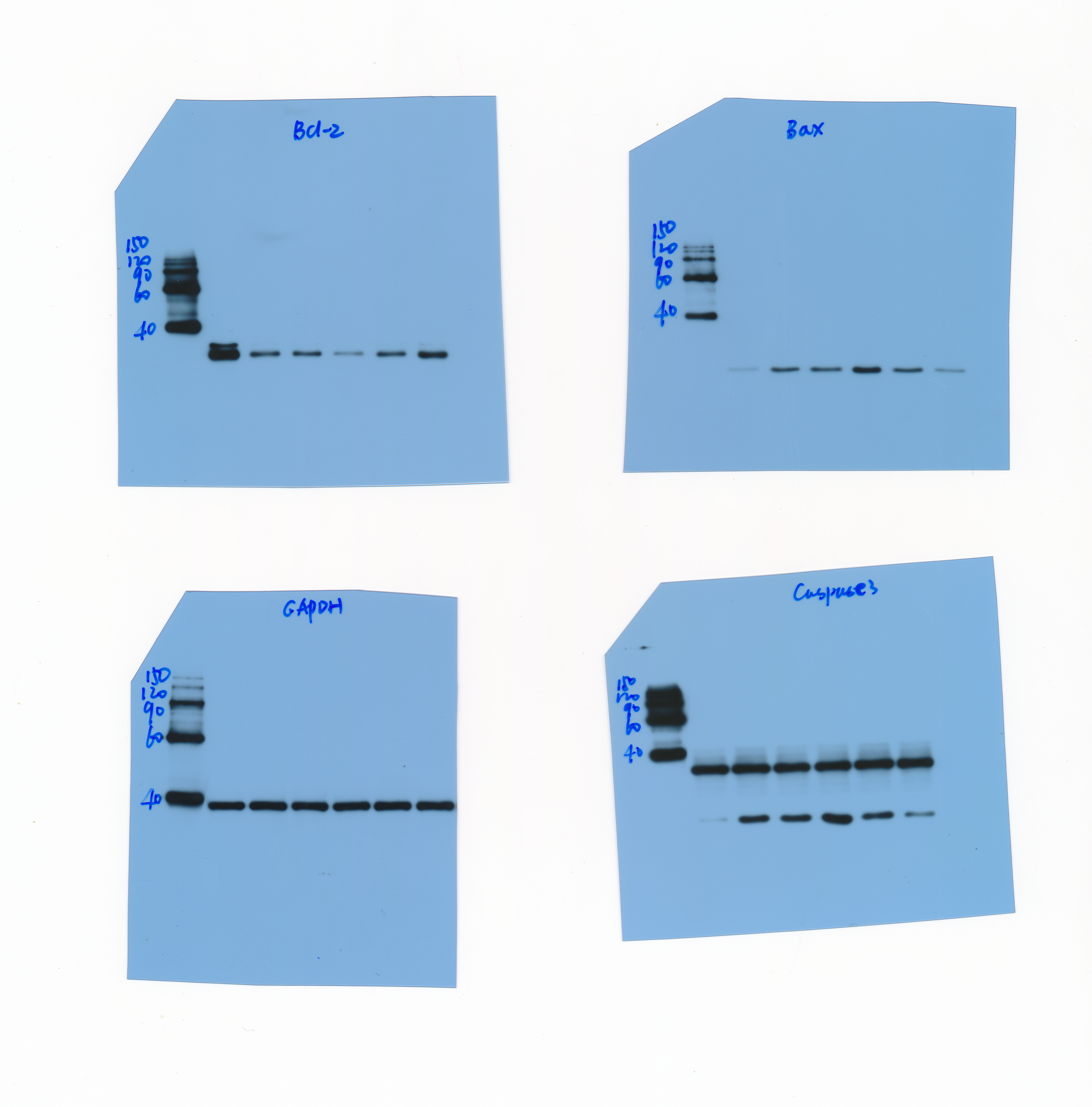

Supplement: Supplementary file 1 — Supplementary Information 1. [file 41598_2024_66433_MOESM1_ESM.tif]

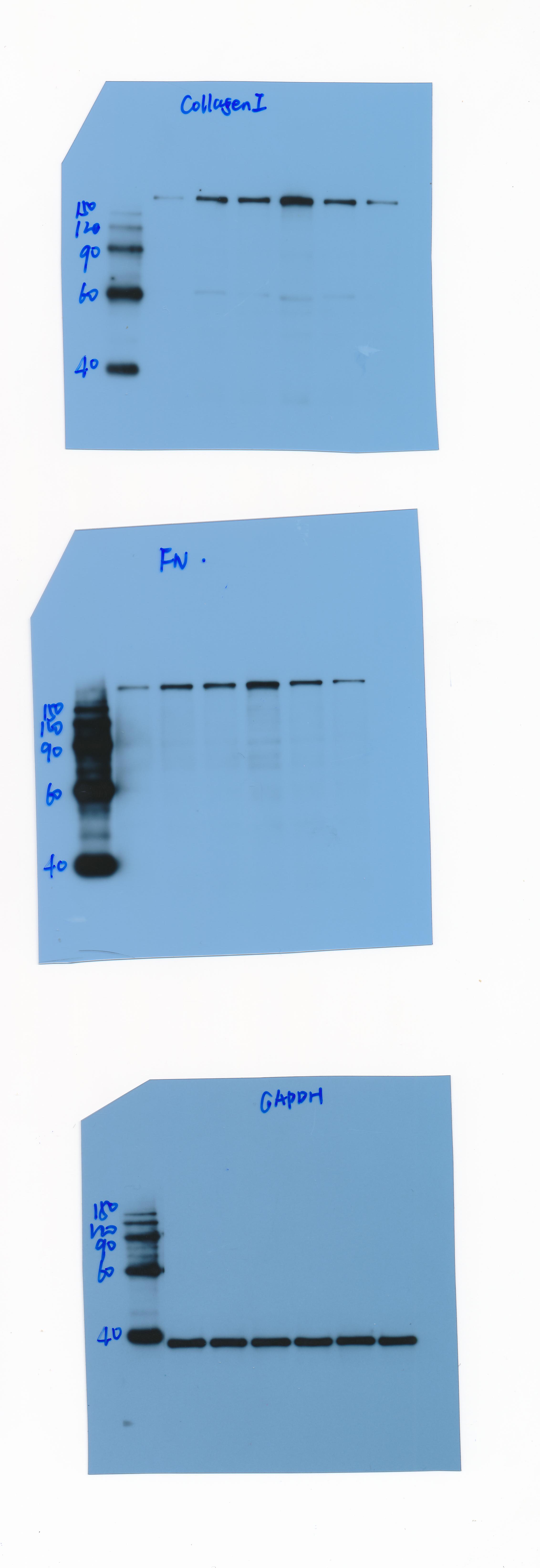

Supplement: Supplementary file 2 — Supplementary Information 2. [file 41598_2024_66433_MOESM2_ESM.tif]

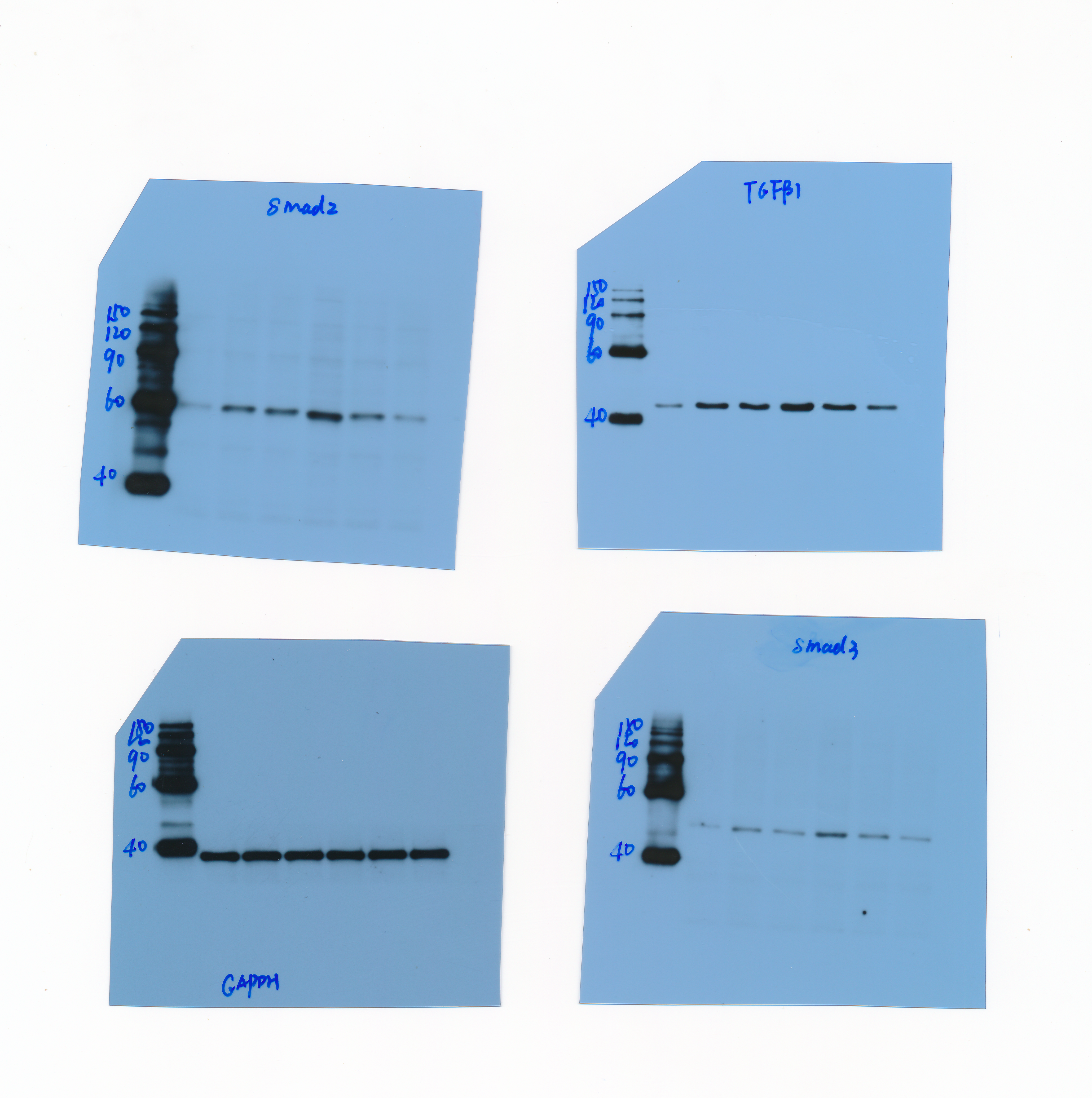

Supplement: Supplementary file 3 — Supplementary Information 3. [file 41598_2024_66433_MOESM3_ESM.tif]
